# Medial prefrontal cortex stimulation modulates the processing of conditioned fear

**DOI:** 10.3389/fnbeh.2014.00044

**Published:** 2014-02-18

**Authors:** Anne Guhn, Thomas Dresler, Marta Andreatta, Laura D. Müller, Tim Hahn, Sara V. Tupak, Thomas Polak, Jürgen Deckert, Martin J. Herrmann

**Affiliations:** ^1^Department of Psychiatry, Psychosomatics and Psychotherapy, University of WürzburgWürzburg, Germany; ^2^Department of Psychiatry and Psychotherapy, University of TübingenTübingen, Germany; ^3^LEAD Graduate School, University of TuebingenTuebingen, Germany; ^4^Department of Psychology, University of WürzburgWürzburg, Germany; ^5^Department of Cognitive Psychology II, University of Frankfurt/MainFrankfurt, Germany; ^6^Institute of Medical Psychology and Systems Neuroscience, University of MünsterMünster, Germany

**Keywords:** fear conditioning, memory consolidation and extinction, learning, transcranial magnetic stimulation (TMS), medial prefrontal cortex (mPFC)

## Abstract

The extinction of conditioned fear depends on an efficient interplay between the amygdala and the medial prefrontal cortex (mPFC). In rats, high-frequency electrical mPFC stimulation has been shown to improve extinction by means of a reduction of amygdala activity. However, so far it is unclear whether stimulation of homologues regions in humans might have similar beneficial effects. Healthy volunteers received one session of either active or sham repetitive transcranial magnetic stimulation (rTMS) covering the mPFC while undergoing a 2-day fear conditioning and extinction paradigm. Repetitive TMS was applied offline after fear acquisition in which one of two faces (CS+ but not CS−) was associated with an aversive scream (UCS). Immediate extinction learning (day 1) and extinction recall (day 2) were conducted without UCS delivery. Conditioned responses (CR) were assessed in a multimodal approach using fear-potentiated startle (FPS), skin conductance responses (SCR), functional near-infrared spectroscopy (fNIRS), and self-report scales. Consistent with the hypothesis of a modulated processing of conditioned fear after high-frequency rTMS, the active group showed a reduced CS+/CS− discrimination during extinction learning as evident in FPS as well as in SCR and arousal ratings. FPS responses to CS+ further showed a linear decrement throughout both extinction sessions. This study describes the first experimental approach of influencing conditioned fear by using rTMS and can thus be a basis for future studies investigating a complementation of mPFC stimulation to cognitive behavioral therapy (CBT).

## Introduction

The extinction of conditioned fear describes the decrement of conditioned responses (CR) after repeatedly presenting a formerly conditioned stimulus (CS) that no longer predicts an unconditioned stimulus (UCS). Extinction learning, memory consolidation and recall of extinction memory have been found to represent different stages of the extinction process, which is also supported by a distinct cortico-limbic functionality (Quirk and Mueller, 2008). At the beginning of the extinction learning, the amygdala shows a profound activation increase to the CS which decreases throughout extinction learning while ventro medial prefrontal cortex (vmPFC) activation meanwhile increases. This reversed amygdala-vmPFC correlation has been shown to reduce the expression of the conditioned fear response. Heightened vmPFC activation thereby inhibits the amygdala's expression of fear during successful extinction recall, i.e., when the already consolidated extinction memory is retrieved (Etkin et al., 2011; Linnman et al., 2012). VmPFC contribution thus appears to be a precondition for sufficient consolidation and later recall extinction memory in animals (Quirk and Mueller, 2008) as well as in humans (Phelps et al., 2004; Kalisch et al., 2006).

Due to homologous prefrontal structures in the rodent and human brain (Milad and Quirk, 2012), results obtained from fear-conditioned animals can be transferred to fear modulation in humans. This is of interest since deficient fear modulation is seen in patients suffering from anxiety disorders (e.g., see Bremner et al., 2005; Milad et al., 2009). A meta-analysis verified that patients with anxiety disorders generally show stronger CR during extinction relative to healthy controls (Lissek et al., 2005). This appears to be caused by a failure of consolidating and recalling extinction memory that most likely originates from a mPFC dysfunction (Rauch et al., 2006; Etkin, 2012).

Since exposure therapy as an effective treatment for anxiety disorders (Foa, 2006) represents the implementation of extinction, it is of clinical relevance to improve extinction learning and extinction memory consolidation. In this regard, manipulations of memory consolidation processes have been established in cross-species translational research. Pharmacologically, D-cycloserine (DCS), a partial N-methyl-D-aspartic acid (NMDA) agonist, has been shown to facilitate fear extinction in rats (Walker et al., 2002; Ledgerwood et al., 2005), which initiated the usage of DCS to augment exposure therapy in patients with anxiety disorders (e.g., Ressler et al., 2004). Acute DCS administration during symptom provocation has been shown to increase prefrontal cortex activity in phobic patients (Aupperle et al., 2009) confirming the reported mPFC dysfunction in anxiety disorders. However, the additional beneficial effects of DCS are rather small when provided in combination with an effective treatment such as cognitive behavioral therapy (CBT; Siegmund et al., 2011) Thus, DCS is suggested to be exclusively indicated for treating severely impaired patients (Siegmund et al., 2011; Klumpers et al., 2012). Moreover, experimental conditioning studies in healthy volunteers failed to show benefits of DCS on extinction learning or extinction recall (Guastella et al., 2007; Klumpers et al., 2012) thereby contradicting the above mentioned animal results (e.g., Walker et al., 2002). A different strategy to improve fear extinction is to electrically stimulate prefrontal regions involved in extinction memory consolidation. In this regard, Milad and Quirk (2002) demonstrated a facilitated extinction in rats that underwent high-frequency stimulation of the infralimbic cortex (IL)—the homolog of the vmPFC in the rat brain. Compared to non-stimulated controls, these rats showed immediate CR attenuation during extinction learning, which persisted to an extinction recall test conducted 24 h later (see also Kim et al., 2010). This inhibitory effect of IL stimulation was ascribed to a reduced responsiveness of output neurons in the central amygdala (Quirk et al., 2003). Thus, electrical stimulation of mPFC structures in rats facilitated extinction learning and extinction recall. So far, it is unclear whether stimulation of homologous regions in humans could have likewise beneficial effects. In this regard, transcranial magnetic stimulation (TMS) represents a suitable method for the translation from animal to human studies (Etkin, 2012).

TMS is a non-invasive technique for stimulating the human cerebral cortex using a brief high-current pulse applied via an electromagnetic coil placed above the scalp (Hallett, 2000). Depending on the stimulation parameters the produced magnetic field can either inhibit (<1 Hz) or excite (>5 Hz) a focal cortical area, most likely by inducing changes in synaptic plasticity linked to learning and memory (Hoogendam et al., 2010). TMS in its repetitive form (rTMS) is able to produce effects beyond the time of stimulation and exceeding the targeted area (Ilmoniemi et al., 1997; Guse et al., 2010). Baeken et al. (2010) investigated one session of 10 Hz rTMS applied to the right dorsolateral prefrontal cortex (dlPFC) in healthy volunteers while passively viewing emotional faces. They found a significant attenuation of right amygdala activation when evaluating negatively valenced stimuli. The use of rTMS as a method to facilitate extinction has been already proposed a decade ago (Milad and Quirk, 2002), but was not accomplished so far.

The aim of the present study was to investigate whether high-frequency rTMS (10 Hz) can modulate the processing of conditioned fear. Based on the results of a previous study in which mPFC contribution during extinction learning was measured with functional near-infrared spectroscopy (fNIRS), a prefrontal cluster depicting increased mPFC activation during extinction learning was targeted (Guhn et al., 2012). Compared to a sham stimulated control group, active stimulation was expected to diminish CR expression during extinction learning and extinction recall due to an increased mPFC activation. In order to verify a rTMS influence on several levels, we implemented different dependent variables indexing conditioned fear, i.e., fear-potentiated startle (FPS) and skin conductance responses (SCR) as psychophysiological measures, fNIRS as an index of neural activity as well as self-reports representing learning on a conscious level. The results of this study could be the basis for investigating the adjunct impact of rTMS to CBT in patients with anxiety disorders.

## Materials and methods

### Subjects

Eighty-eight healthy, TMS-naïve volunteers (43 men) were recruited from a large sample collected at a Collaborative Research Center (SFB-TRR 58) of the Universities in Münster, Würzburg and Hamburg, Germany, as well as internet announcements. They were screened for current mental health and right-handedness by using the Mini International Neuropsychiatric Interview (M.I.N.I., Sheehan et al., 1998) and the Edinburgh Inventory (Oldfield, 1971). All female volunteers were additionally screened for a regular menstrual cycle and the non-usage of any hormonal contraceptives for at least 3 months prior to measurement. In order to account for facilitating effects of estrogen on extinction learning (Glover et al., 2012) and extinction recall (Milad et al., 2010; Zeidan et al., 2011), women only participated in the experiment during their early follicular phase (defined as the first 5 days of a 28-day cycle) when estradiol and progesterone levels are low. Contraindications regarding the TMS safety guidelines (Wassermann, 1998) such as epilepsy, use of pacemakers or pregnancy were assured. Participants gave written informed consent in accordance with the Declaration of Helsinki in its latest version from 2008. All procedures were approved by the ethics committee of the University of Würzburg.

Three female subjects dropped out due to the experience of discomfort while receiving TMS application and were thus not considered for further data analysis. Demographic data of the remaining *N* = 85 participants are presented in Table 1. None of the reported variables reached statistical significance for group comparisons between active and sham TMS (student's *t*-test, *p* > 0.05). Group differences can therefore be interpreted in terms of TMS effects.

**Table 1:** **Sample description**.

		**Active group**	**Sham group**
Sex	Males	21	22
	Females	19	23
Age	*M* ± *SD*	23.9 ± 3.0	24.6 ± 4.5
Education (years)	*M* ± *SD*	12.7 ± 0.6	12.9 ± 0.4
UCS intensity^a^	(0–10)	6.5 ± 1.6	6.3 ± 2.1
STAI^b^	Trait	36.8 ± 6.8	33.9 ± 7.1
	State	36.7 ± 6.9	36.1 ± 9.1
PANAS^c^ I	Positive affect	2.95 ± 0.5	3.08± 0.5
	Negative affect	1.23 ± 0.3	1.20 ± 0.2
PANAS^c^ II	Positive affect	2.70 ± 0.6	2.68 ± 0.6
	Negative affect	1.17 ± 0.3	1.22 ± 0.3
*N*		40	45

a UCS intensity determined the subjective level of aversiveness of the scream used as unconditioned stimulus (UCS) on a scale ranging from 0 for “not unpleasant” to 10 for “extremely unpleasant.”

b State-Trait Anxiety Inventory (Laux et al., 1981).

c Positive and Negative Affect Scale (Krohne et al., 1996), I indicate the first investigation before the experiment, II the second investigation after completing study day 1.

### Design

**Figure 1:**
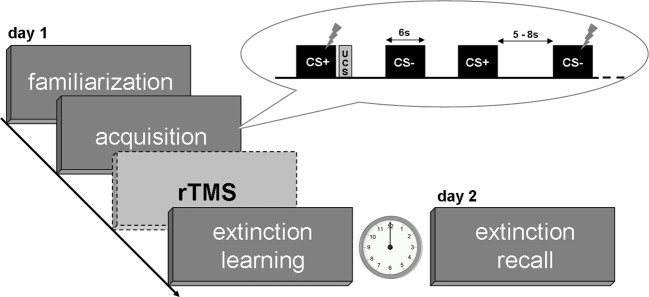
**Experimental design**. Flashes indicate startle stimuli during CS presentations as primary measure of the conditioned response.

The paradigm consisted of four phases divided into familiarization, fear acquisition and extinction learning on day 1 and a test for extinction recall on day 2 (see Figure 1). Two male neutral faces served as conditioned stimuli (CS; Tottenham et al., 2009) and an aversive scream of 95 dB served as unconditioned stimulus (UCS; IADS, Bradley and Lang, 1999). Volunteers were first familiarized with both CS by presenting each face eight times without the UCS. During the following fear acquisition phase consisting of 32 CS presentations one neutral face (CS+) was randomly followed by the UCS in 50% of trials whereas the other face (CS−) never preceded the UCS. Both extinction phases (day 1 and 2) consisted of 40 trials in total (20 CS+, 20 CS−) without UCS presentations. CS stimuli were presented for 6000 ms duration separated by jittered inter trial intervals (ITI) of 5000–8000 ms displaying a fixation cross. The UCS lasted 1380 ms and followed CS+ offset after a jittered temporal gap of 0–1000 ms (Guhn et al., 2012). The assignment of CS+ and CS− was counterbalanced across subjects and stimuli were presented in a pseudo-randomized order such that maximally three similar faces followed each other. Presentation® version 12.2 software (Neurobehavioral Systems, Inc., Albany, CA, USA) was used for presenting the paradigm.

### Repetitive transcranial magnetic stimulation (rTMS)

Following the fear acquisition phase, subjects received one offline session of either active or placebo (sham) rTMS prior to performing extinction learning on day 1. Stimulation was applied via a round coil (MMC-140 Parabolic) of a Medtronic MagPro X100 stimulator (Medtronic MagPro, Düsseldorf, Germany) to a cluster within the medial prefrontal cortex. The coil was positioned in the middle of the cluster which was identified by marking channel 26 of the NIRS probe set corresponding to the MNI coordinates *x* = 14.5, *y* = 68.3, *z* = 21.3 (according to http://www.jichi.ac.jp/brainlab/virtual_registration/Result3x11_E.html. This channel represents the center of the mPFC activation cluster for which we found an increase in oxygenated hemoglobin concentration over the time course of extinction learning in a prior study (Guhn et al., 2012). Inter-subject variance was considered by assigning Fpz according to the 10-20 EEG system (Jasper, 1958). Emanating from Fpz, channel 26 was marked resulting in slightly varying positions for TMS coil positioning based on the participants individual head sizes. The upper edge of the coil was tilted 2 cm away from the scalp in order not to stimulate the premotor cortex; the handle of the coil was pointed upwards. The rTMS protocol was adapted from Baeken et al. [2010; stimulation intensity of 110% of the individual resting motor threshold (RMT), 10 Hz stimulation frequency, 40 trains of 4 s duration (1560 pulses), inter train intervals of 26 s], who found an amygdala attenuation in response to negative stimuli after one rTMS session. For the present study, this protocol was selected corroborating the intention that it should impact the fear circuit in the same way, i.e., the proposed increased prefrontal top–down modulation of subcortical systems, in particular the amygdala. Sham rTMS was applied using a placebo coil (MC-P-B70 Placebo) which appeared similar in placement and acoustic properties to the active coil but had a magnetic shield embedded limiting the amount of the magnetic field. In order to control for the proposed facilitatory effects of active rTMS, fNIRS was used to monitor blood oxygenation as an index of functional brain activity in the mPFC directly following the stimulation, i.e., during extinction learning, and during extinction recall on day 2 (see below). The TMS protocol and the subsequent attachment of the NIRS probeset resulted in a time lag of approximately 25 min between the fear acquisition and extinction learning phase.

### Procedure

On the day of stimulation (day 1), subjects were first familiarized with the experimental design and asked to answer questionnaires concerning mood (Positive and Negative Affect Scale, PANAS; Krohne et al., 1996) and anxiety (State-Trait Anxiety Inventory, STAI; Laux et al., 1981). Subsequently, they were introduced to the TMS machine by identifying the individual RMT defined as the lowest stimulation intensity capable of inducing a visible finger movement at least 5 times out of 10 single pulses over the right hand area of the primary motor cortex. TMS application and all measurements were conducted in a sound-attenuated, electrically shielded and air-conditioned cabin. Subjects were prepared for the experiment by attaching headphones and electrodes for startle potentiation and skin conductance recordings (see below). They were instructed about the separation of the experiment into three parts: (1) in the first half of the experiment they are confronted with two neutral faces on the computer screen as well as two auditory sounds (familiarization and fear acquisition), (2) subsequently the rTMS application to their forehead while sitting still on a chair, and (3) immediately after the stimulation the second half of the experiment again consisting of faces and auditory stimuli (extinction learning). Subjects were not instructed about the CS+/UCS contingency or the UCS absence during the extinction phase. At the end of day 1 the PANAS was assessed a second time to evaluate a potential rTMS impact on mood (Tupak et al., 2013).

On day 2, subjects had to answer a self-construed questionnaire concerning rTMS side effects based on Wassermann (1998) (“Did you experience any adverse side effects after the rTMS yesterday? If yes, please mark which kind of discomfort you experienced and how long it lasted.”). They were prepared for physiological recordings and underwent the test for extinction recall while the instruction resembled that of day 1. TMS was not applied a second time. Afterwards subjects were unblinded to the rTMS condition and were paid for participation.

Conditioned fear responses (CR) were assessed by FPS, SCR, fNIRS, and subjective valence and arousal ratings for CS+ and CS−.

### Fear-potentiated startle (FPS)

The eyeblink component of the startle reflex was measured by recording electromyographic (EMG) activation of the right orbicularis oculi muscle. Two 5 mm Ag/AgCl disc surface electrodes were positioned approximately 1 cm below the pupil and 1 cm below the lateral canthus of the right eye (impedance <5 kΩ). A third electrode was placed at the right mastoid and served as isolated ground. The acoustic startle stimuli consisted of a 50 ms burst of white noise with 40 ms plateau and 5 ms rise and fall time at intensities of 100 dB (sound pressure level, SPL) delivered binaurally via in-ear headphones. No background sound was presented. Startle probes were delivered in half of the trials (4000 ms after CS onset) and ITI (randomly between 3000 and 5000 ms). EMG activity was recorded via a 72-channel amplifier (QuickAmp, Brain Products GmbH, Munich, Germany) and sampled at 1000 Hz. Data was acquired, saved and analyzed with Vision Recorder/Analyzer Version 2.0 (Brain Products GmbH, Munich, Germany). The EMG-signal was filtered with a 28 Hz high-pass and a 500 Hz low-pass filter (time constant 0.0057 s, 24 dB per octave). A notch filter was applied to control for components caused by (electro-)magnetic interference. After rectification signals were smoothed using a 50 ms moving average filter. Each segment was baseline-corrected 50 ms prior to the startle probe onset. Startle amplitudes were further defined as peak magnitudes (in microvolt) from the corrected EMG signal between 21 and 200 ms following probe onset. Artifact rejection was performed manually for every single peak. Startle non-responders on either one or both days were identified by mean magnitudes of less than 5 μV per day and excluded accordingly (*n* = 14). Another male subject had to be excluded due to a nystagmus, which made startle blink recording impossible. In order to allow for inter-individual differences, absolute blink magnitudes were normalized using *z*-standardization (Blumenthal et al., 2005). ITI startle probes were further utilized as control condition for CS+ and CS− by converting startle magnitudes during each CS presentation (X) into *Z* scores using the ITI mean and standard deviation per phase (*Z_CS_* = (*X_CS_* – *M*_ITI_)/*SD*_ITI_); (e.g., Bonnet et al., 1995; Blumenthal et al., 2005).

### Skin conductance response (SCR)

SCR was assessed by using two Ag/AgCl electrodes attached to the thenar eminence of the subjects' left palm. Measurements were acquired via a 72-channel amplifier and a Galvanic Skin Response (GSR) sensor which constantly delivered a 0.5 V current (Brain Products GmbH, Munich, Germany). The sampling rate was set to 1000 Hz. SCR recording and analyses were performed with Vision Recorder/Analyzer Version 2.0 (Brain Products GmbH, Munich, Germany). Offline, raw data were first high-pass filtered with 1 Hz and a notch filter of 50 Hz and afterwards segmented into CS+ and CS− trials that were baseline-corrected 1000 ms prior to CS onset. SCR were characterized by peak responses in a time window of 1 to 5 s after CS onset. Artifact rejection was performed manually for every single trial. Similarly to the FPS analyses SCR data were z-transformed across both days without the first four respective CS trials in order to account for inter-individual differences. Six non-responders had to be excluded and were thus not considered for further analysis.

### Functional near-infrared spectroscopy (fNIRS)

Functional NIRS is based on near-infrared light of different wave lengths that is emitted to the cortical surface by means of sensors attached to the participant's forehead and thereby measures local changes of blood oxygenation. A detailed description can be found elsewhere (Obrig and Villringer, 2003). Oxygenation concentration was measured with the continuous wave system ETG-4000 (Hitachi Medical Co., Tokyo, Japan) using a 3 × 11 array which covered the prefrontal cortex. The interoptode distance was set to 3 cm. Signals were acquired with a sampling rate of 10 Hz. The method was included in order to discuss FPS, SCR, and rating results in the light of rTMS induced mPFC activation within the targeted fNIRS channels. We hypothesized that if rTMS modulates the processing of conditioned fear, it will correlate with higher mPFC activation in the cluster for which we found a signal increase from early to late extinction learning in a previous study (Guhn et al., 2012). Accordingly, we time-locked the onset of the signal to the jitter mean, i.e., 6500 ms after CS onset, and manually screened for artifacts due to head movement or technical problems. Signals were further processed by applying a cosine filter of 0.5 Hz correcting for low-frequency signal drifts. The four regressors (CS+ early, CS+ late, CS− early, CS− late) were modeled as delta functions and convolved with a gaussian hemodynamic response function at 6.5 s peak time. Time series for blood oxygenation (O_2_Hb) during both extinction sessions were then assessed by applying a general linear model approach. Beta estimates for stimulus (CS+, CS−) by phase mean (extinction learning early, extinction learning late, extinction recall early, extinction recall late) between groups (active, sham) were investigated by using repeated measures analyses of variance (ANOVA).

### Subjective ratings

Subjective CS+ and CS− ratings were assessed through self-assessment manikins (SAM; Bradley and Lang, 1994) for valence and arousal at different time points during the experiment: after familiarization and twice during/after fear acquisition, as well as during/after both extinction sessions. Subjects were asked to indicate whether a face was perceived as pleasant or unpleasant and whether it induced arousal or not on a 9-point Likert Scale.

### Statistical analysis

Demographic data such as age and years of education were compared between groups with student *t*-tests. Psychometric data (UCS-intensity, PANAS, and STAI scores) were analyzed by using the Mann–Whitney-*U*-test, rTMS side effects by using Fisher's Exact Probability Test.

For FPS and SCR analyses, subjects were first characterized by CS+ and CS− responses during the acquisition phase. We analyzed paired (CS-UCS) as well as unpaired (CS-noUCS) CS+ trials since UCS followed the CS with a short temporal gap, i.e., the analyzed segment did not include the actual UCS delivery. Subjects who did not show higher responses for CS+ than CS− were not considered for further TMS group comparisons due to non-successful fear conditioning (e.g., Phelps et al., 2004). Likewise 22 subjects (13 women) had to be excluded. Potential group differences on a descriptive or psychometric level (age, UCS-intensity, STAI-T, STAI-S; PANAS) were accounted for and did not reveal any significant results. CS trials were averaged for each stimulus (CS+, CS−) per phase (acquisition, extinction learning, extinction recall) and statistically evaluated using repeated measures ANOVA with stimulus and phase mean as within-subject factors and group (active, sham) as between-subject factor. A *p*-value < 0.05 was considered significant; Greenhouse Geisser correction was applied in case of non-sphericity. *Post-hoc t*-tests were used when (1) stimulus × phase × group interactions proved to be significant or (2) stimulus × phase interactions proved to be significant without significant group effects; in the second case *post-hoc t-tests* were conducted within groups. Additionally, we analyzed gender effects for FPS and SCR data and tested for significant interactions between gender and TMS group. A short theoretical background and discussion of these results is provided in the supplement.

## Results

### Fear potentiated startle (FPS)

The final sample consisted of *n* = 21 (13 women) subjects receiving active and *n* = 24 subjects (12 women) receiving sham stimulation. ANOVA revealed significant main effects for stimulus [*F*_(1, 43)_ = 15.35, *p* < 0.001], the interaction of stimulus × phase [*F*_(1.5, 66.5)_ = 5.7, *p* = 0.009] and a trend-wise significant stimulus × phase × group interaction [*F*_(1.5, 66.5)_ = 2.92, *p* = 0.074].

**Figure 2:**
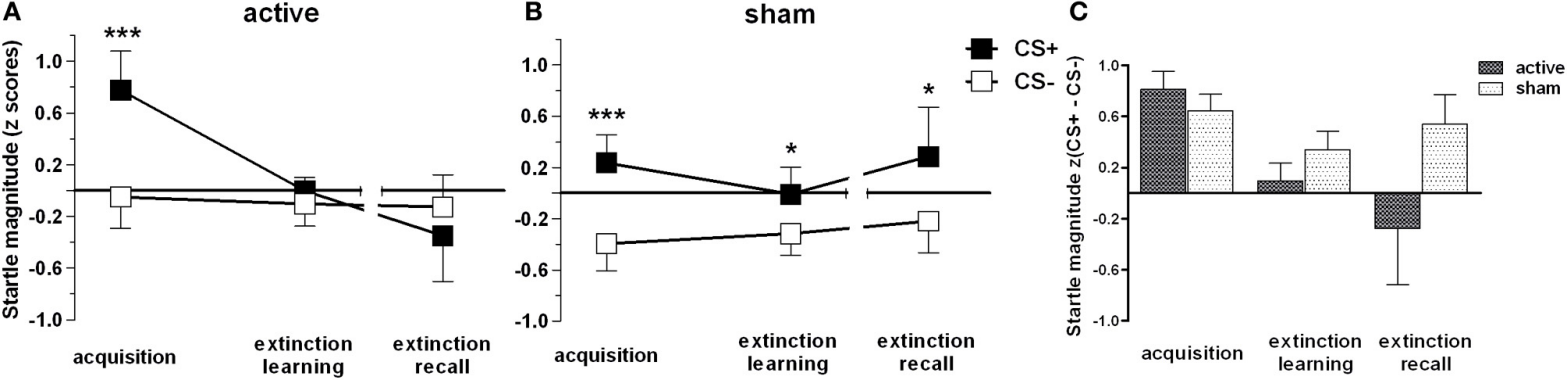
**Fear-potentiated startle magnitudes for CS+ and CS− trials for active (A) and placebo (B) group and the difference score (C) accordingly**. In all experimental phases mean responses and standard errors of the mean (SEM) are depicted. Asterisks indicate significant differences (^*^p < 0.05, ^***^p < 0.001). **(C)** illustrates CS+ and CS− trials as difference scores to indicate that groups did not differ in their conditioned response during the acquisition phase [independent *t*-contrast: *t*_(43)_ = 1.47, *p* > 0.05].

**Figure 3:**
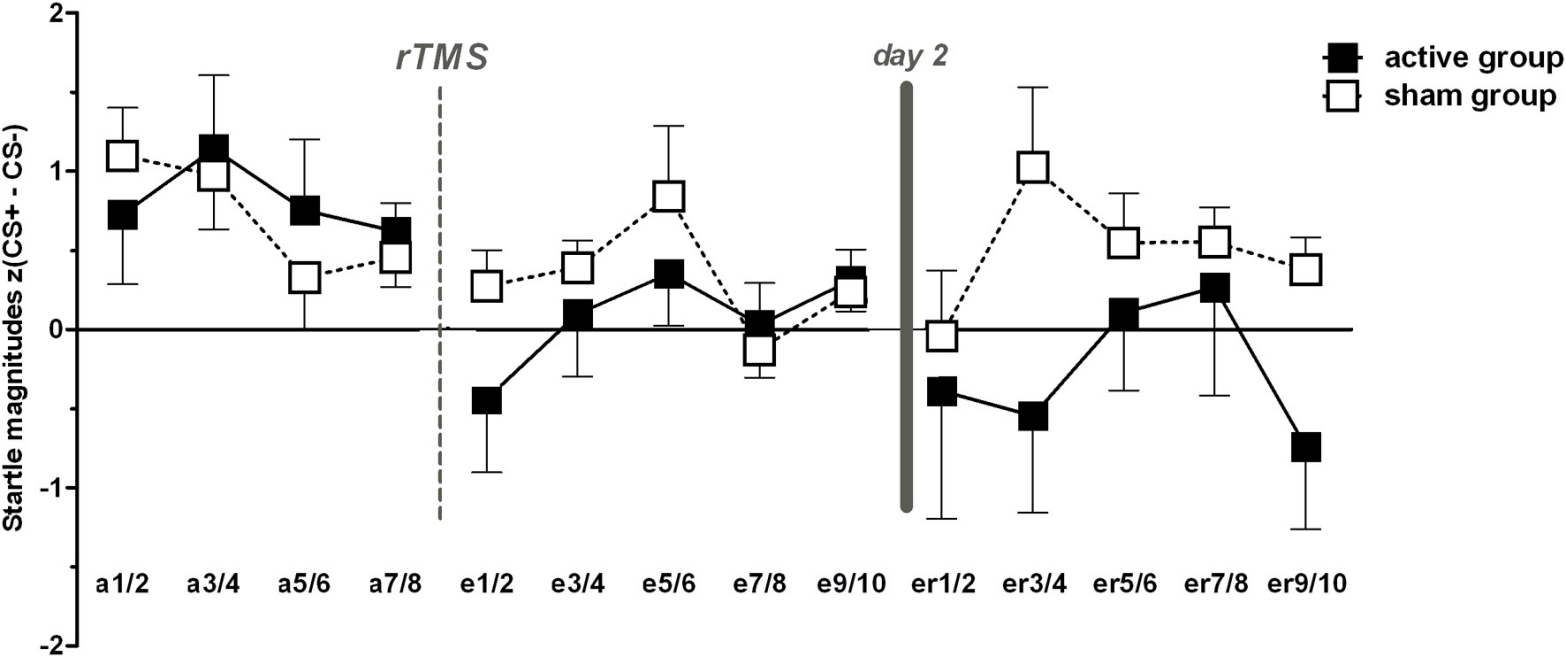
**Conditioned responses (*CS+ minus CS−*) indexed by fear potentiated startle magnitudes for acquisition (a), extinction (e), and extinction recall (er)**. For reasons of clarity two trials were averaged for each phase, respectively. Note that in the middle of each phase online valence and arousal ratings were conducted. Error bars indicate the standard error of the mean (SEM).

**Figure 4:**
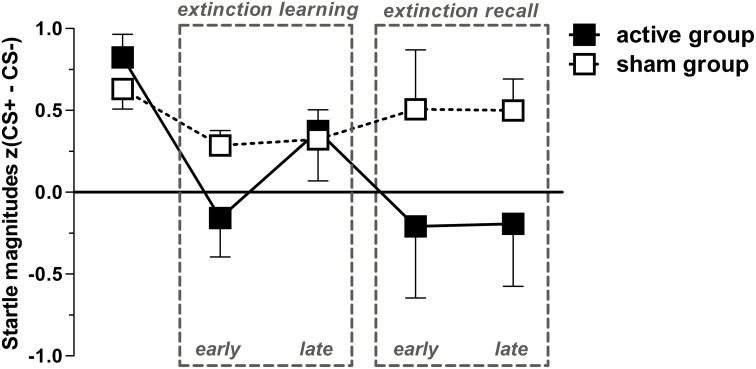
**Conditioned responses (*CS+ minus CS−*) for both groups indexed by Fear-potentiated startle magnitudes (mean + SEM)**. The active group exhibit a significant linear trend through both extinction phases indicated by a proportionately CR decrement from fear acquisition (first data point) throughout both extinction phases. For the active group, all dependent *t*-tests (*p*_one-tailed_ < 0.05) for acquisition with each extinction phase showed significant results, except for acquisition vs. late extinction day 1 which revealed only a trend-wise significant *p*-value (*p* < 0.1). For the sham group, only the early extinction day 1 compared to the acquisition phase revealed a significant difference thereby indicating extinction learning; all other scores resemble the acquisition phase (*p* > 0.05).

As expected, *t*-tests revealed significant differences between CS+ and CS− trials during acquisition within both groups (*p* < 0.001), but revealed sustained CS+/CS− discrimination for sham only, i.e., higher FPS responses for CS+ than for CS− for both extinction learning [*t*_(23)_ = 2.3, *p* = 0.031] and extinction recall [*t*_(23)_ = 2.44, *p* = 0.023; Figure 2]. CR for both groups in time course are provided in Figure 3. In order to statistically analyze these group differences during the experimental phases we continued to separate each extinction session into an early and a late phase consisting of 10 trials each for which we used the CS+/CS− differences. A one-way ANOVA examining the effects of phase (acquisition, early extinction learning day 1, late extinction learning day 1, early extinction recall day 2, late extinction recall day 2) on FPS magnitudes revealed a trend-wise significant main effect of phase for the active group [*F*_(2.3, 46.7)_ = 2.98, *p* = 0.054]. This is composed of a negative linear trend [*F*_(1, 20)_ = 4.19, *p* = 0.054]: FPS responses decreased proportionately through all phases while the sham group neither showed a significant main effect of phase (*p* > 0.79) nor significant trends. Figure 4 shows the time course of the difference scores (CS+ minus CS−) throughout the five phases.

### Skin conductance response (SCR)

**Figure 5:**
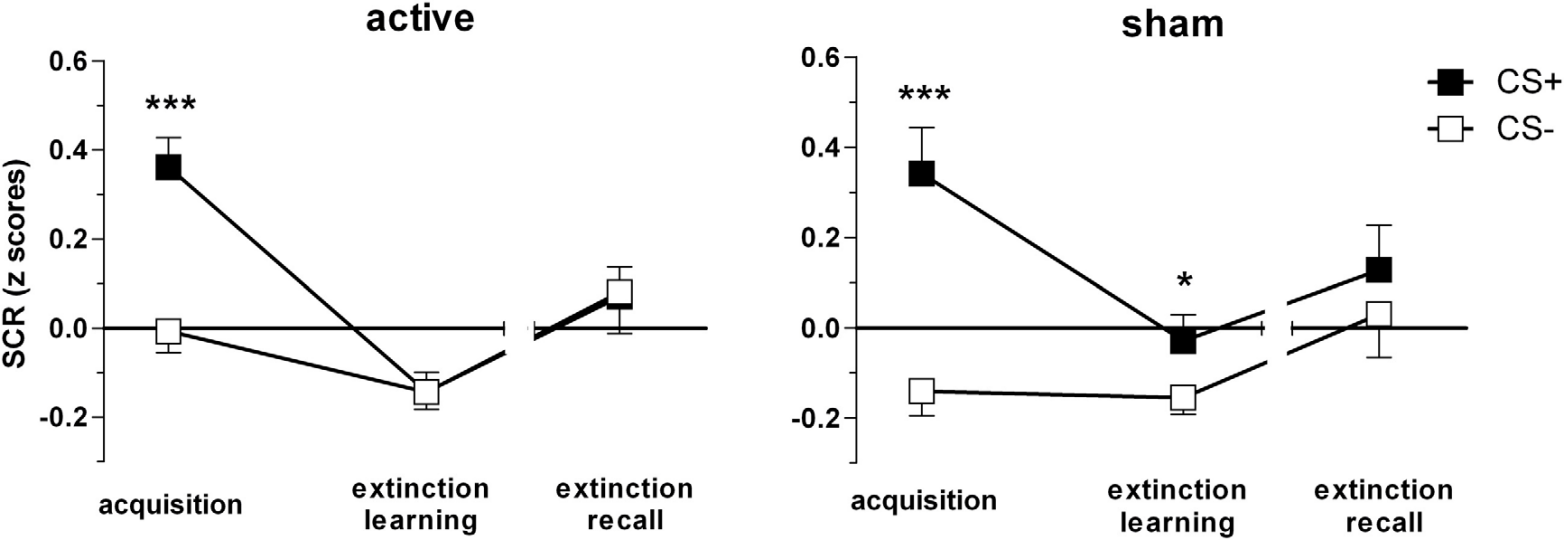
**Skin conductance responses (SCR) for CS+ and CS− trials during acquisition, extinction learning on day 1, and extinction recall on day 2, per group, respectively**. Depicted are means and standard errors of the mean. Asterisks indicate significant differences (^*^p < 0.05, ^***^p < 0.001).

The final sample for SCR analyses consisted of 47 subjects, *n* = 26 active (15 women) vs. *n* = 21 sham group (9 women). The three-way ANOVA revealed significant stimulus [*F*_(1, 45)_ = 26.28, *p* < 0.001], phase [*F*_(1.6, 74)_ = 7.62, *p* = 0.001] and stimulus × phase interaction effects [*F*_(2, 90)_ = 14.84, *p* < 0.001]. Group did not influence main or interaction effects (*p* > 0.1). Both groups showed successful discrimination during acquisition (*p* < 0.001). Notably, the sham group still showed the CS+/CS− discrimination sustained during extinction learning [*t*_(20)_ = 2.11, *p* = 0.047] while the active group displayed no significant CS+/CS− differences (*p* > 0.9; Figure 5).

### Subjective ratings

In order to keep the sample constant we examined self-reports only for subjects who were analyzed either for FPS or SCR data (*n* = 62, see Table 2). This sample did not differ from the non-conditioners (*n* = 23) in any of the assessed descriptive or psychometric measures.

**Table 2:** **Subsample of successful conditioned volunteers for data analysis of the subjective ratings**.

		**Active group**	**Sham group**
Sex	Males	15	16
	Females	17	14
Age	*M* ± *SD*	23.81 ± 3.2	24.43 ± 3.5
UCS intensity	(0–10)	6.23 ± 1.6	6.47 ± 2
STAI	Trait	36.84 ± 6.9	34.5 ± 7.68
	State	37 ± 7.35	37.3 ± 10.4
*N*		32	30

Successful conditioning was defined by a higher CR on CS+ vs. CS− trials during the fear acquisition phase. None of the reported variables reached statistical significance for group comparisons.

All subjects indicated successful fear acquisition as evident from significant main effects for stimulus [valence: *F*_(1, 60)_ = 8.16, *p* = 0.006; arousal: *F*_(1, 60)_ = 27, *p* < 0.001], phase [valence: *F*_(2.6, 157.7)_ = 18.31, *p* < 0.001; arousal: *F*_(3, 180)_ = 42.22 *p* < 0.001] and significant stimulus × phase interactions [valence: *F*_(2.3, 137.7)_ = 11.8, *p* < 0.001; arousal: *F*_(1.9, 114)_ = 15.67, *p* < 0.001]. CS+ and CS− were equally evaluated during familiarization [valence: *t*_(61)_ = 0.27, *p* = 0.790; arousal: *t*_(61)_ = −0.18, *p* = 0.857] but self-reports diverged significantly during fear acquisition, in that CS+ was rated as more unpleasant [*t*_(61)_ = 4.79, *p* < 0.001] and evoked higher arousal [*t*_(61)_ = −5.57, *p* < 0.001] than CS−. This significant discrimination persisted over both extinction learning [valence: *t*_(61)_ = 2.05, *p* = 0.044; arousal: *t*_(61)_ = −4.76, *p* < 0.001] and extinction recall [valence: *t*_(61)_ = 2.33, *p* = 0.023, arousal: *t*_(61)_ = −4.19, *p* < 0.001] although CS+ valence increased [*t*_(61)_ = −6.88, *p* < 0.001] and CS+ arousal decreased in the course from acquisition to extinction [*t*_(61)_ = 7.67, *p* < 0.001] again resulting in familiarization-like levels (*p* > 0.1).

**Figure 6:**
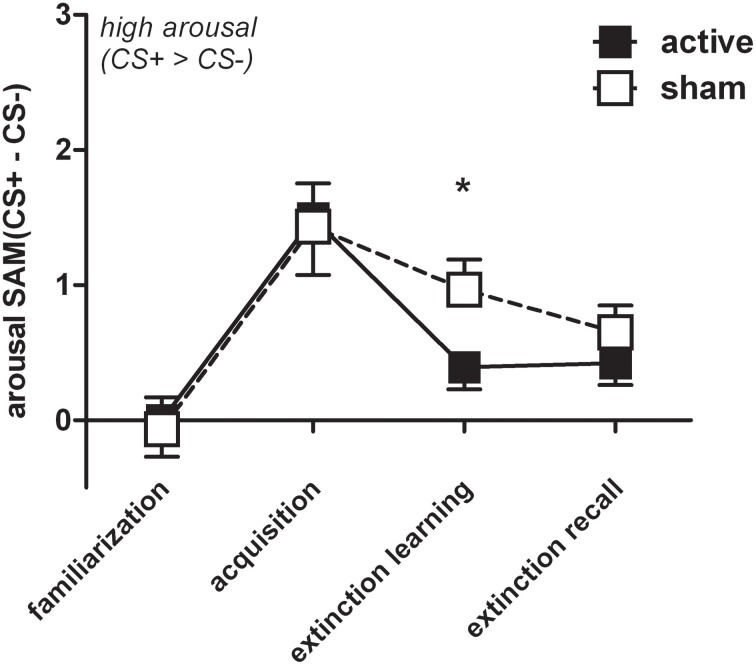
**For reasons of visualization arousal difference scores (*CS+ minus CS−*) were depicted (M + SEM) during familiarization, acquisition, extinction learning, and extinction recall for the active (*n* = 32) and the sham group (*n* = 30)**. Groups significantly differed during extinction learning (^*^p < 0.05), i.e., sham showed higher arousal for CS+ than CS− trials.

In order to account for rTMS induced group differences we conducted a three-way ANOVA examining effects of stimulus by phase with only two levels (extinction learning, extinction recall) between groups. We found a significant stimulus × phase × group interaction for arousal [*F*_(1, 60)_ = 4.33, *p* = 0.042]. The active group (*n* = 32) discriminated significantly less between CS+ and CS− while the sham group (*n* = 30) persisted to evaluate CS+ as more arousing than CS− [*t*_(53.8)_ = −2.01, *p* = 0.043] resembling the FPS and SCR results (Figure 6). Valence ratings revealed no group differences. The three-fold interaction did not reach statistical significance (*p* > 0.2) for the whole sample (*N* = 85), including conditioners *and* non-conditioners.

### Functional near-infrared spectroscopy (fNIRS)

**Figure 7:**
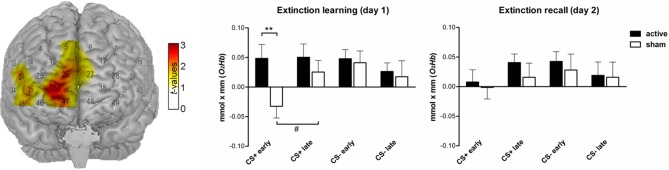
**Functional NIRS results (O_2_Hb) during both extinction phases (*n* = 25). Left**: T-map superimposed on a standard brain. During the early extinction learning the active group showed a higher signal for CS+ than the sham group in a cluster of 10 probeset channels covering the medial prefrontal cortex. The bar charts in the middle and on the right depict the corresponding beta estimates for CS+ and CS− trials (^**^p_one-tailed_ < 0.01). The sham group showed a trend-wise significant (^#^*p*_one-tailed_ < 0.1) signal increase from early to late extinction learning in response to CS+ trials while the active group persisted to show a high concentration level.

We neither found significant group differences during extinction learning nor during extinction recall in the sample of *n* = 62 which was used for the subjective ratings. Exploratorily we analyzed the subsample of volunteers fulfilling the requirements for the analysis of both FPS *and* SCR (*n* = 12 active and *n* = 13 sham, two data sets were not included into the analysis due to an insufficient signal quality) since those participants were believed to have the strongest conditioning response regarding the consistency across measurements. However, we are well aware that the results have to be regarded cautiously. For the cluster reported in our pilot study (10 medial prefrontal channels expanding to the right hemisphere: 5, 16, 24, 26, 27, 35, 36, 37, 45, 47) the active and sham group differed in the amount of O_2_Hb in response to CS+ during the early extinction learning phase for which the active group displayed a higher signal than the sham group [student *t*-test: *t*_(23)_ = 2.65, *p*_one−tailed_ = 0.008]. While there was no signal change from the early to the late phase in the active group, the sham group showed a trend-wise significant signal increase [*t*_(23)_ = −1.61, *p*_one−tailed_ = 0.067] resembling the signal increase reported in the previous study. During the extinction recall on day 2 there were no within or between-group differences (see Figure 7).

### Side effects

Side effects were assessed using a questionnaire which contained previously published rTMS side effects such as headache, neck pain, dizziness, drowsiness, nausea, speech, or sleep problems, problems to concentrate, paraesthesia, seizures, muscle contraction, faint, local discomfort at the stimulated site and ear noise (Wassermann, 1998). Subjects were asked to evaluate these side effects in their intensity and duration before unblinding them regarding the TMS group. For completeness, the *n* = 3 females who dropped out due to rTMS discomfort were included in the analysis (*N* = 88). Overall, rTMS was well tolerated. Twenty-two subjects (25%) reported side effects, therefrom 10 subjects of the sham group. Type of side effects per group are depicted in Table 3, no other side effects were quoted. Headaches as the most prominent side effect lasted less than 1 h in 11 subjects; 5 subjects complained about headaches for less than 6 h and 2 for less than 12 h. There was neither a significant group difference concerning the overall frequency of side effects nor the type of side effects (*p* > 0.49), except for neck pain which was trend-wise quoted more frequently by the sham group (*p* = 0.056). Altogether, the results demonstrate that subjects were actually TMS-naïve.

**Table 3:** **Frequencies of quoted rTMS side effects**.

	**Active group (*n* = 43)**	**Sham group (*n* = 45)**
Headaches	9	9
Neck pain	0	5
Drowsiness	1	2
Problems to concentrate	0	2
Local discomfort (forehead)	2	3

Possible mood changes caused by rTMS were evaluated using PANAS × group repeated measures ANOVA. Positive affect showed a significant main effect [*F*_(1, 86)_ = 45.94, *p* < 0.001] indicating that subjects rated their affect prior to the experiment as more positive than afterwards. Negative affect did not change. The group interaction did not reach statistical significance, i.e., rTMS did neither induce negative nor positive mood changes (*p* > 0.1).

## Discussion

In the present study, one session of high-frequency rTMS was applied to the mPFC in healthy, TMS-naïve subjects who underwent a 2-day discriminative fear conditioning and extinction paradigm. In order to increase a top-down regulation of the mPFC thereby modulating the processing of conditioned fear, facilitatory rTMS was administered offline before an extinction learning phase. Consistent with our hypothesis, the active group displayed diminished CS+/CS− discrimination during extinction learning (day 1) as evident from FPS data and to a smaller extend from SCR as well as from subjective arousal ratings. Moreover, rTMS had a persisting effect on extinction recall (day 2) as seen with FPS while the sham group revealed higher conditioned fear responses to CS+ than to CS− trials and reported higher arousal for CS+ during extinction learning. This study describes the first experimental approach of influencing conditioned fear by using rTMS.

Resembling the animal data of prefrontal electrical stimulation (Milad and Quirk, 2002; Kim et al., 2010), we found significant group differences for active vs. sham stimulation during extinction learning (FPS, SCR, and arousal ratings) and extinction recall (FPS). While IL stimulation studies in rats revealed the most prominent results during extinction recall, such a *sustained* effect of rTMS in the present study was limited to the FPS data. Hereby the CS+ responses linearly declined from high FPS magnitudes during acquisition to low magnitudes during late extinction recall without the prominent fear return typically emerging when subjects are confronted with the former CS+ a day after the extinction learning (Bouton, 2002). Quirk et al. (2003) provided a probable explanation for likewise results by showing that mPFC stimulation in animals inhibited central amygdala output neurons and thereby reduced the conditioned fear. In this regard, the here applied active mPFC stimulation should have increased the activity of amygdaloid intercalated cells, resembling a top–down mechanism (Quirk and Beer, 2006; Milad et al., 2007). With regard to the startle response which is mediated by a neural pathway that directly originates from the amygdala (Davis et al., 1997), the improved extinction recall as indexed by the FPS data could thus represent attenuated amygdala activation. This interpretation of our findings is consistent with results of amygdala attenuation following dlPFC stimulation while processing negative pictures using the same rTMS protocol (Baeken et al., 2010). Moreover, the results that we obtained for fNIRS point toward higher O_2_HB values for the active compared to the sham group which confirms the interpretation of increased mPFC activity through rTMS.

Based on these experimental results in healthy volunteers, rTMS might be a promising complementing therapeutic tool in anxiety patients when combined with exposure therapy, which is based on the principles of extinction learning and extinction memory recall. Pathological anxiety and even anxiety-related personality traits in healthy subjects have been associated with hyper-reactivity of the conditioned amygdala response and deficient prefrontal recruitment. An impaired inhibition of the amygdala through the mPFC is hereby suggested to cause enhanced vulnerability to pathological anxiety and risk for relapse (Sehlmeyer et al., 2011). According to the present results, rTMS in combination with exposure therapy might effectively inhibit the amygdala response via an increased prefrontal cortex activity. As mentioned before, the pharmacological intervention with DCS was able to increase PFC activity in phobic patients while it was surprisingly unable to show facilitation effects on experimental fear extinction in healthy subjects (Guastella et al., 2007; Klumpers et al., 2012). Therefore, it is most likely that the present rTMS effect on extinction memory would be even more marked in patients with anxiety disorders showing overall heightened fear reactions and diminished fear extinction.

The exact underlying neurophyisological mechanisms of rTMS remain unclear. Hallett (2000) and Hoogendam et al. (2010) propose that rTMS influences the consolidation of learning by modifying excitatory synaptic efficacy or neuronal synchrony. By comparing high- and low-frequency stimulation in mice using an offline approach, successful extinction learning was associated with long-term potentiation (LTP) while long-term depression (LTD) resulted in the return of conditioned fear (Herry and Garcia, 2002). High-frequency rTMS over 10 consecutive days in rats was further associated with a lasting increase of prelimbic levels of the brain-derived neurotrophic factor (BDNF), a neuroplasticity marker involved in LTP (Gersner et al., 2011). Thus, in the present study rTMS might have either promoted prefrontal LTP during extinction learning as well or interfered with LTP during the consolidation of the fear memory. In order to enlighten which learning phase was actually modulated, future studies should consider a 3-day design in order to be able to separate fear acquisition and extinction learning into consecutive days. Thereby, the memory stage which is influenced by rTMS, i.e., fear or extinction memory could be disentangled.

The present study has a number of limitations which need to be considered when interpreting the results. First of all, the comparability with findings of animal studies regarding the mPFC-amygdala interplay is limited by the fact that the present design used an offline rather than an online TMS approach in which the stimulation is applied time-locked to CS presentations (Milad and Quirk, 2002). However, an offline TMS approach enabled us to assure that participants indeed exposed themselves to the magnetic field. Prefrontal TMS affects face muscles which commonly irritates TMS-naïve participants at the beginning. Therefore, in an online approach participants might avoid the stimulation in case of discomfort by moving the head slightly away from the coil. Instead of an online stimulation a TMS protocol inducing long lasting effects up to 30 min was selected (George et al., 1996). The TMS coil positioning in the present study was further not identical to the electrical IL stimulation in the rat studies. Due to the coil size, the limited stimulation depth to the cortex and the high stimulation intensity the vmPFC as homologues region to the IL was not selected as rTMS target region. According to a pilot study a more dorsal part of the mPFC was referred to as target region since this region was associated with an increased activity to CS+ trials in an extinction learning session (Guhn et al., 2012). In order to proof the targeted mPFC region, inhibiting the mPFC via low-frequency rTMS should result in prohibited or at least decelerated fear extinction (Herry and Garcia, 2002) which future studies should confirm.

With regard to the data analysis it has to be further mentioned that the number of volunteers who showed higher CR to CS+ than to CS− after the fear acquisition phase was limited regarding the whole sample. This was the result of a methodological challenge we had to face: In contrast to anxiety patients, healthy volunteers exhibit a fast and efficient extinction learning and extinction recall (e.g., see Milad et al., 2008, 2009). In order to resemble deficient extinction learning, the extinction process had to be decelerated. This was achieved by reducing the CS+/UCS pairings during the fear acquisition phase. The UCS in average only followed every second presentation of the CS+ (50% reinforcement rate) and thereby became a less predictable signal for UCS occurrence leading to a prolonged resistance to extinguish the CS+. Investigating interventions on extinction learning in healthy participants raise the question of how to establish optimal circumstances in which an intervention such as rTMS can show advantages. While we constituted decelerated extinction in healthy participants, we had to face the problem of non-conditioners not adapting to the danger signaling properties of the CS+ and/or the safety signaling properties of the CS−. Based on findings by Van Well et al. (2012) we therefore decided to exclude these participants accepting a higher number of non-considered data sets. Comparing neural substrates between conditioners and non-conditioners in an instructed fear paradigm (reinforcement rate 75%), Van Well et al. found significant group differences in stimulus differentiation between CS+ and CS− as well as differential stimulus peak activations within the amygdala and other regions. This shows that conditioners exhibited higher peak activations for CS+ compared to CS−. Furthermore, amygdala activation significantly correlated with FPS and thereby supports FPS as reliable and specific index of fear. Assuming that rTMS interacts with memory consolidation via the mPFC-amygdala top-down regulation, our hypothesis could not have been tested in participants who did not established fear, which was defined as a positive CS+/CS− discrimination during fear acquisition.

In conclusion, our results indicate that rTMS provides a non-invasive and well-tolerated therapeutic tool as evidenced by the low frequency of side effects which can modulate the processing of conditioned fear in healthy human subjects. Therefore it can serve as a basis for future studies investigating the precise learning stage, i.e., fear vs. extinction memory and its respective causal mechanisms. Additionally future studies can use these results to investigate the effect of rTMS on fear extinction in patients with anxiety disorders as well as its proposed beneficial effect in combination with psychotherapy.

### Conflict of interest statement

The authors declare that the research was conducted in the absence of any commercial or financial relationships that could be construed as a potential conflict of interest.
