# Supplementary material for: Medial prefrontal cortex stimulation modulates the processing of conditioned fear
Source: Front Behav Neurosci. 2014 Feb 18;8:44. doi: 10.3389/fnbeh.2014.00044 (PMC3927128; doi:10.3389/fnbeh.2014.00044)
Supplement: Supplementary file 1 [file DataSheet1.PDF]

Supplemental Information

Medial prefrontal cortex stimulation modulates the processing of conditioned fear

Anne Guhn, Thomas Dresler, Marta Andreatta, Laura D. Müller, Tim Hahn, Sara V. Tupak, Thomas Polak, Jürgen Deckert, Martin J. Herrmann

Supplemental Data

Gender effects

Epidemiological studies agree on the fact, that women are more susceptible to develop mood and anxiety disorders (Kessler et al., 2005). The incidence of all anxiety disorders such as posttraumatic stress disorder, panic disorder, agoraphobia, social anxiety, and phobias is two to three times higher for women compared to men (Seeman, 1997). Interestingly, gender differences arise starting with the reproductive years of women suggesting that sex hormones play an important role in the development and maintenance of anxiety disorders (Seeman, 1997).

To date, the number of publications controlling for or manipulating the hormonal status in females is quiet low (Farrell et al., 2013). Existing studies however do point towards an important role of the estrogen level which varies throughout the menstrual cycle i.e. being low at the beginning and high at the end of the follicular phase (mid cycle). Rat studies comparing females in these two phases of the estrous cycle in a classical pavolovian fear conditioning study revealed differences particularly during extinction recall (Milad et al., 2009;Zeidan et al., 2011): Females in the late follicular phase showed a comparable extinction recall than males while females in the early follicular phase exhibited an impaired performance. Translational studies in humans revealed comparable findings (Milad et al., 2010) and further point to specific gender effects in brain regions correlating with emotional learning (Zeidan et al., 2011). Due to the fact that the amygdala and the ventromedial prefrontal cortex (vmPFC) contain a relatively high number of estrogen receptors (Goldstein et al., 2001), the neural reactivity at these sites, which are involved in the stress response (Goldstein et al., 2010) and more precisely in fear extinction (Merz et al., 2010;Zeidan et al., 2011), is modulated by estrogen. Thus, the investigation of sex hormones enables the possibility to adjust therapies for women depending on their current state within the menstrual cycle. Since the present investigation focuses on the effects of repetitive transcranial magnetic stimulation (rTMS) on extinction learning and extinction recall in premenopausal naturally cycling women, gender effects will be considered for the data analysis. All women were recruited in the early follicular phase which was defined as the first five days of a regular menstrual cycle. Additionally, they did not take oral contraceptives for at least three month prior to the measurement. Based on the existing literature, low estrogen levels are hypothesized to cause deficits in extinction recall. Therefore, a possible compensating rTMS effect is tested.

Statistically, gender was integrated as a second between-subject factor in a stimulus (CS+, CS-) x phase (acquisition, extinction learning, extinction recall) by group (active, sham) repeated measurements ANOVA. The resulting sample distributions with regard to the dependent variables FPS and SCR are depicted in Table 1.

Table S1. Sample distributions for FPS (left) and SCR data (right) with regards to the TMS group (active, placebo) and gender (male, female).

FPS	♂	♀	
**active group**	8	13	21
**placebo group**	12	12	24
	20	25	

SCR	♂	♀	
**active group**	11	15	26
**placebo group**	12	9	21
	23	24	

FPS: Fear-potentiated startle response; SCR: Skin conductance response

For the *FPS data*, the ANOVA revealed a significant interaction between gender and stimulus [$F(1,41) = 13.24$, $p \leq .001$], which resulted from a significantly higher conditioned response in women compared to men during the fear acquisition phase [$t(40.2) = 2.12$, $p = .04$]; no gender differences were observed for both extinction phases. Moreover, group and gender did not interact significantly. On a descriptive level, both genders show lower conditioned responses in the active group than in the placebo group (Figure S1).

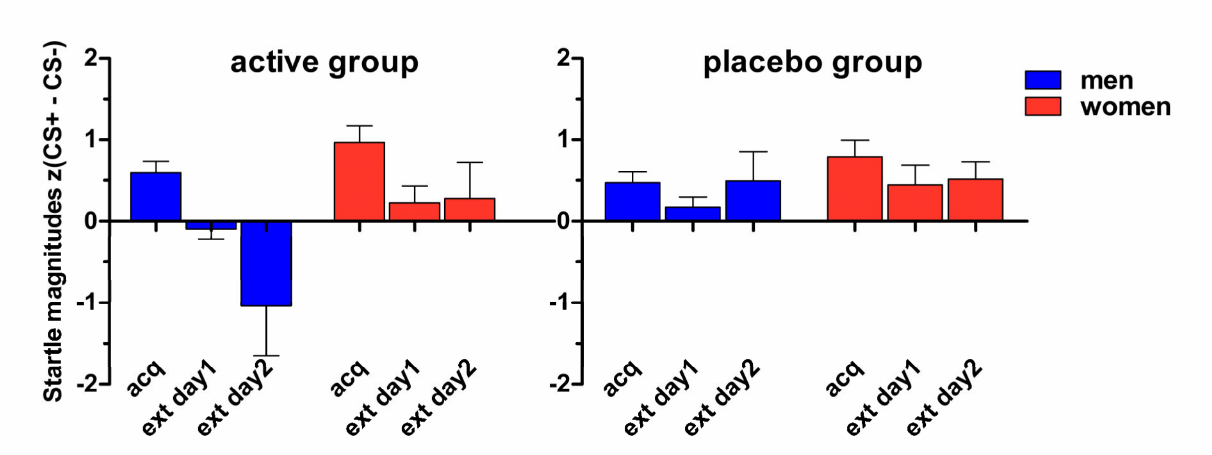

Fig. S1. Conditioned responses (CS+ minus CS-) indexed for fear potentiated startle magnitudes in men and women divided for TMS group (active, placebo). Depicted are phase means for acquisition (acq), extinction learning (ext day1) and extinction recall (ext day2); error bars indicate the standard error of the mean (SEM).

For the *SCR data*, adding the gender to the analysis resulted in a significant interaction for stimulus x phase x group x gender [$F(2,86) = 4.42$, $p < .05$]. In order to elucidate this four-fold interaction we divided the sample into two subsamples for men and women and calculated a stimulus x phase by group ANOVA respectively (for results see Table S2). Both subsamples showed the expected stimulus x phase interaction representing successful fear conditioning and fear extinction, however only in women this interaction was trend-wise impacted by TMS stimulation. Thus, while women seem to benefit from a prefrontal stimulation, stimulation group did not reveal a significant interaction for men since the descriptive illustration points towards a successful recall of extinction memory even without stimulation (Figure S2). However, post-hoc tests did not confirm the facilitated extinction learning and extinction recall statistically in women statistically [$t(22) < 1.3$, $p > .1$], probably due to the low statistical power.

Table S2. SCR results for a stimuli x phase x group ANOVA, analyzed for men and women respectively.

gender	factor	F	df	p
men	stimulus	14.17	1, 21	.001
	phase	7.1	2, 42	.002
	stimulus x phase	10.69	2, 42	<.001
	stimulus x phase x group	2.0	2, 42	.149
women	stimulus	11.65	1, 22	.002
	phase	5.24	2, 44	.009
	stimulus x phase	3.93	2, 44	.027
	stimulus x phase x group	2.43	2, 44	.099

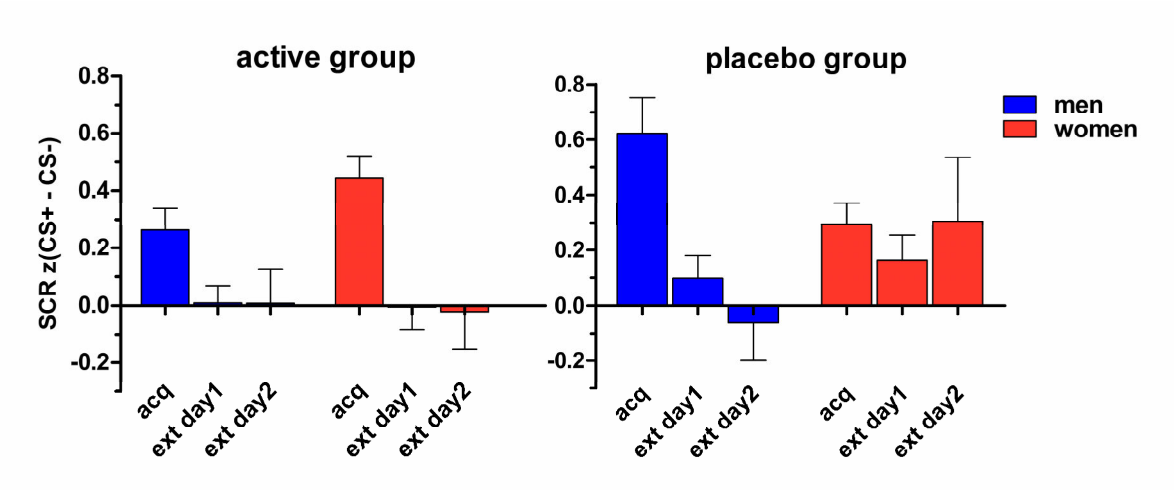

Fig. S2: Conditioned responses (CS+ minus CS-) indexed for skin conductance responses (SCR) in men and women divided for TMS group (active, placebo). Depicted are phase means for acquisition (acq), extinction learning (ext day1) and extinction recall (ext day2); error bars indicate the SEM.

To summarize, the analyses of gender effects revealed opposing results for FPS and SCR data. While there was no significant interaction between gender and TMS session for FPS data, the consideration of gender seemed to improve the TMS impact on the SCR data by showing an additional profit for women. Thereby, the SCR results are in line with the facilitating role of estrogen on extinction recall (Zeidan et al., 2011): In the placebo group, women show a numerically larger conditioned response during extinction recall, which is consistent with an impaired recall of the safety memory. Therefore, TMS might constitute a treatment option which could compensate for the reduced prefrontal top-down control in early cycle women when estradiol is low. However, given that we did not find a significant group x gender interaction in the FPS data, which was the main outcome variable of the present study, the SCR results have to be regarded with caution and necessarily has to get replicated in an independent and numerously larger sample.

Nonetheless, when considering rTMS as a possible treatment option the simultaneous consideration of gender and hormonal status in women might be promising. Exposure therapy as the clinical application of fear extinction for instance could be scheduled when the estrogen level is high. However, if this is not possible, exposure therapy could be combined with rTMS in order to compensate for low estrogen, for instance in women taking oral contraceptives which reduces endogenous cycling estradiol levels.

References

Farrell, M.R., Sengelaub, D.R., and Wellman, C.L. (2013). Sex differences and chronic stress effects on the neural circuitry underlying fear conditioning and extinction. *Physiology & Behavior*.

Goldstein, J.M., Jerram, M., Abbs, B., Whitfield-Gabrieli, S., and Makris, N. (2010). Sex differences in stress response circuitry activation dependent on female hormonal cycle. *J Neurosci* 30**,** 431-438.

Goldstein, J.M., Seidman, L.J., Horton, N.J., Makris, N., Kennedy, D.N., Caviness, V.S., Faraone, S.V., and Tsuang, M.T. (2001). Normal Sexual Dimorphism of the Adult Human Brain Assessed by In Vivo Magnetic Resonance Imaging. *Cerebral Cortex* 11**,** 490-497.

Kessler, R.C., Berglund, P., Demler, O., Jin, R., Merikangas, K.R., and Walters, E.E. (2005). LIfetime prevalence and age-of-onset distributions of dsm-iv disorders in the national comorbidity survey replication. *Archives of General Psychiatry* 62**,** 593-602.

Merz, C.J., Tabbert, K., Schweckendiek, J., Klucken, T., Vaitl, D., Stark, R., and Wolf, O.T. (2010). Investigating the impact of sex and cortisol on implicit fear conditioning with fMRI. *Psychoneuroendocrinology* 35**,** 33-46.

Milad, M.R., Igoe, S.A., Lebron-Milad, K., and Novales, J.E. (2009). Estrous cycle phase and gonadal hormones influence conditioned fear extinction. *Neuroscience* 164**,** 887-895.

Milad, M.R., Zeidan, M.A., Contero, A., Pitman, R.K., Klibanski, A., Rauch, S.L., and Goldstein, J.M. (2010). The influence of gonadal hormones on conditioned fear extinction in healthy humans. *Neuroscience* 168**,** 652-658.

Seeman, M.V. (1997). Psychopathology in women and men: focus on female hormones. *Am J Psychiatry* 154**,** 1641-1647.

Zeidan, M.A., Igoe, S.A., Linnman, C., Vitalo, A., Levine, J.B., Klibanski, A., Goldstein, J.M., and Milad, M.R. (2011). Estradiol modulates medial prefrontal cortex and amygdala activity during fear extinction in women and female rats. *Biological Psychiatry* 70**,** 920-927.
